# A Phospho-SIM in the Antiviral Protein PML is Required for Its Recruitment to HSV-1 Genomes

**DOI:** 10.3390/cells3041131

**Published:** 2014-12-10

**Authors:** Miles C. Smith, Andrew C. Box, Jeffrey S. Haug, William S. Lane, David J. Davido

**Affiliations:** 1 Department of Molecular Biosciences, University of Kansas, Lawrence, KS 66045, USA; 2 Stowers Institute for Medical Research Flow Cytometry Facility, Kansas City, MO 64110, USA; E-Mails: ACB@stowers.org (A.C.B.); JSH@stowers.org (J.S.H.); 3 Mass Spectrometry and Proteomics Resource Laboratory, Harvard University, Cambridge, MA 02138, USA; E-Mail: wlane@harvard.edu; 4 Current address: Department of Microbiology and Physiological Systems, University of Massachusetts Medical School, Worcester, MA 01655, USA; E-Mail: milesc.smith@umassmed.edu

**Keywords:** PML, ND10, PML-NB, phosphorylation, HSV, ICP0, intrinsic immunity

## Abstract

Herpes simplex virus type 1 (HSV-1) is a significant human pathogen that infects a large portion of the human population. Cells deploy a variety of defenses to limit the extent to which the virus can replicate. One such factor is the promyelocytic leukemia (PML) protein, the nucleating and organizing factor of nuclear domain 10 (ND10). PML responds to a number of stimuli and is implicated in intrinsic and innate cellular antiviral defenses against HSV-1. While the role of PML in a number of cellular pathways is controlled by post-translational modifications, the effects of phosphorylation on its antiviral activity toward HSV-1 have been largely unexplored. Consequently, we mapped phosphorylation sites on PML, mutated these and other known phosphorylation sites on PML isoform I (PML-I), and examined their effects on a number of PML’s activities. Our results show that phosphorylation at most sites on PML-I is dispensable for the formation of ND10s and colocalization between PML-I and the HSV-1 regulatory protein, ICP0, which antagonizes PML-I function. However, inhibiting phosphorylation at sites near the SUMO-interaction motif (SIM) of PML-I impairs its ability to respond to HSV-1 infection. Overall, our data suggest that PML phosphorylation regulates its antiviral activity against HSV-1.

## 1. Introduction

Herpes simplex virus type 1 (HSV-1) is most well known as the cause of the facial ulcerations commonly referred to as cold sores. It primarily undergoes lytic replication in the orofacial epithelia, where viral gene expression occurs in an ordered cascade consisting of an immediate-early (IE), early (E), and late (L) phase and eventually gives rise to progeny virus that can infect the surrounding area, including the sensory neurons. Once in the sensory neurons, the virus can traffic to the trigeminal ganglia where it establishes a lifelong latent infection. Certain traumatic stimuli that result in a loss of immune surveillance can allow the virus to exit latency and result in a reoccurrence of lytic viral replication. Owing to its relatively benign symptoms and ability to persist latently, HSV-1 is a widespread human pathogen, with upwards of 70%–80% of the population, depending on socioeconomic class, being infected by the virus [1]. While these facial ulcerations are a relatively minor concern from a health perspective, serious complications arise when the virus infects areas outside of its preferred orofacial region, such as the eye or brain where the virus can, respectively, cause scarring and blindness of the eye and a fatal encephalitis. Additionally, an increasing number of genital herpes infections are caused by HSV-1 and may be the primary agent in certain populations [2].

Host cells initially attempt to limit the activities of HSV-1 through intrinsic defense mechanisms. One mediator of the intrinsic antiviral defense is the promyelocytic leukemia protein (PML). PML is a nuclear regulatory protein present in a majority of cell types. While it is constitutively expressed, the PML promoter contains elements that allow for its upregulation in response to activation of the antiviral interferon pathway [3]. The *PML* gene contains nine exons, giving rise to seven major isoforms that all share a common N-terminal set of domains but differ greatly in their C-terminus [4]. PML is capable of extensive interactions with itself and other proteins, especially those that have been modified by one of the small ubiquitin-like modifier (SUMO) proteins [5,6,7], allowing PML to serve as the nucleating constituent of the nuclear suborganelle, nuclear domain 10 (ND10). Current evidence suggests that PML is itself an E3 SUMO ligase [8,9], though its physiological targets are currently unknown. Through its ability to interact with a wide variety of partners, PML plays a role in numerous cellular pathways, such as apoptosis, the DNA damage response, telomere maintenance, stem cell maintenance, transcription, translation, cellular proliferation, differentiation, and antiviral defense; in most cases, PML responds to stress conditions to slow or limit growth [10].

In the absence of certain viral factors, PML has been shown to affect aspects of the HSV-1 life cycle [11,12,13,14]. Upon nuclear entry of viral DNA, preexisting ND10s disassemble and reform near the sites of incoming viral genomes [15,16]. At these sites, certain ND10 members assist in the loading of chromatin on viral DNA and form a shell that prevents the initiation of viral gene expression, presumably by occluding the ability of transcription factors from interacting with viral DNA and initiating transcription [17].

**Figure 1:**
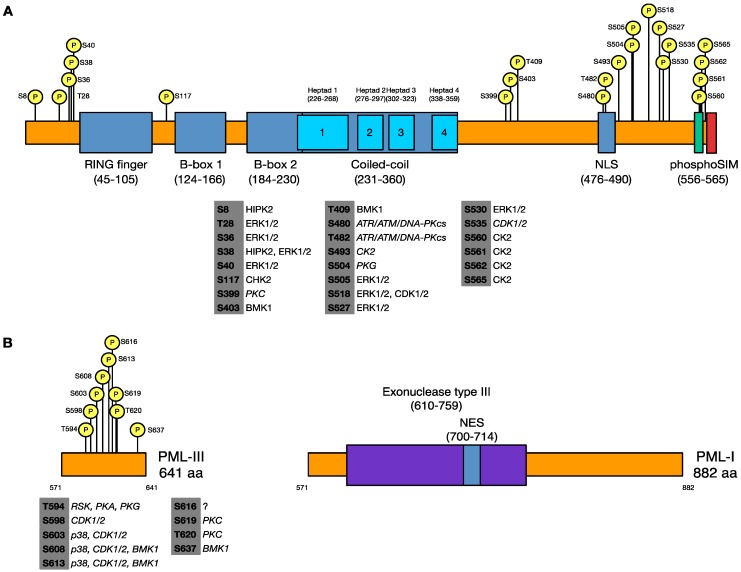
Map of known and novel sites of promyelocytic leukemia (PML) phosphorylation and the kinases that target these residues. (**A**) Sites of phosphorylation from published studies and Table 1. Table below lists cellular kinases that are known sites of phosphorylation or are predicted sites of phosphorylation by either NetPhos 2.0 [33] or PROSITE [34,35] (in italics) to phosphorylate the indicated residues. (**B**) A map of the differing terminal domains of PML isoforms I and III, including phosphorylation sites mapped in the c-terminus of PML-III.

PML is extensively post-translationally modified by SUMOylation, acetylation, ubiquitination, and phosphorylation [18,19]) (Figure 1). These modifications are essential for the activity of PML, and its ability to form ND10s and respond to cellular signals [20]. PML is SUMOylated on at least three lysine residues [21], though additional minor SUMOylation sites have been suggested [22,23]. SUMOylation of PML at its major sites, including K65, K160, and K490, is necessary for proper ND10 formation [6] and exchange of PML between ND10s and the nucleoplasm [24], for partner protein recruitment [25], PML protein stability [26,27,28,29]. PML is phosphorylated on a number of serines and threonines by several cellular kinases, including ERK1/2 [30], p38 [31], BMK1 [32], CK2, CHK2, and HIPK2 (reviewed in [18]). Much as the case with SUMOylation, phosphorylation has a multitude of differing effects on PML activity including altering its stability, localization, and interactions with partner proteins in addition to regulating further post-translational modifications.

Post-translational modifications are known to influence PML’s ability to respond to HSV-1 infection. Shortly upon infection, PML can be found to be recruited to viral genomes in a manner contingent upon its SUMOylation, as forms that cannot be SUMOylated fail to appreciably respond to the nuclear entry of viral DNA and remain positionally stable [36]. Furthermore, these SUMOylation-deficient mutants fail to restrict the ability of HSV-1 mutants that are sensitive to intrinsic defense mechanisms. HSV-1, however, overcomes these defenses through the activity of its E3 ubiquitin ligase, ICP0, which induces the ubiquitination and proteasomal destruction of PML [37,38]. Here again, PML SUMOylation influences the course of infection as ICP0 favors interaction with and degradation of certain SUMOylated forms of PML [38,39]. While SUMOylation of PML is important for its antiviral activity and crosstalk between post-translational modifications, PML SUMOylation can be influenced by phosphorylation [30,40]. Notably, the role of phosphorylation in the control of PML’s antiviral activity, particularly towards HSV-1, has received little attention.

Herein we report that several phosphorylation sites on PML influence its stability in the presence of ICP0 and that mutation of phosphoacceptor sites near its SIM impairs the ability of PML to be recruited to incoming viral genomes. These data support the observation that PML phosphorylation contributes to host defenses.

## 2. Experimental Section

### 2.1. Cells

Human embryonic lung (HEL-299) cells were obtained from the American Type Culture Collection (CCL-137) and were maintained in Minimum Essential Medium Eagle Alpha Modification (αMEM) containing 10% fetal bovine serum (FBS), 2 mM L-glutamine, 10 U/mL penicillin, and 10 U/mL streptomycin. A549 and A549-based cell lines were maintained in Dulbecco’s modified Eagle’s medium containing 5% FCS, 2 mM L-glutamine, 10 U/mL penicillin, and 10 U/mL streptomycin. HepaRG [41], HA-shNeg, HA-shPML [42], and all HA-shPML-derivative cell lines were maintained in William’s E media containing 10% FBS, 2 mM L-glutamine, 10 U/mL penicillin, 0.5 µM hydrocortisone, and 5 µg/mL insulin. Cells transduced with shRNA-encoding lentivirus were kept under antibiotic selection with puromycin at 1 µg/mL; HA-shPML+LNGY, HA-shPML+LNGY-PML.I, and HA-shPML+LNGY-PML.I-derivative cell lines were additionally maintained under selection with G418 at 200 µg/mL.

GP2-293, U2OS, HEp-2 cells and HEp-2 derivative cell lines were maintained in Dulbecco’s modified Eagle’s medium (DMEM) containing 5% FBS, 2 mM L-glutamine, 10 U/mL penicillin, and 10 U/mL streptomycin.

A549 and HEp-2 cells depleted of PML or the luciferase-depleted control line were created by transducing cells with LKO-shPML or LKO-shLuci [13,14] lentiviral stocks using four sequential 1-hour incubations, replacing the previous round of incubation with fresh lentiviral supernate. After the fourth round of transduction, the cells were incubated at 37 °C overnight. The next day, cells were washed three times with PBS and incubated in fresh medium. Two days post-transduction, the cells were placed under selection using puromycin at 1 µg/mL and thereafter maintained in puromycin.

A549 and HepaRG-based cells expressing FLAG- and eCFP-tagged PML were made by transducing cells as above with retrovirus in the presence of 10 µg/mL polybrene as described above.

HepaRG-based cells expressing eYFP-tagged PML were made as described for the creation of shRNA-expressing A549 cells. Two days post transduction, the cells were placed under antibiotic selection using G418 at 800 µg/mL. The resistant outgrowth was expanded before being enriched by fluorescence activated cell sorting as previously described [12].

### 2.2. Plasmids

Vectors encoding the PML ORF were constructed as follows. eCFP was subcloned in the place of eGFP by replacing the NotI-BamHI fragment of pMX-eGFP (a gift from Toshio Kitamura) with that from pECFP-N1 (Clontech). An EcoRI-MfeI fragment containing a myc-tagged PML isoform 4 from the vector pCImycPML-fl [39] was subcloned into the EcoRI site of pMX-eCFP to create pMX+mycPML4+eCFP. A MluI-MfeI fragment of pMX+mycPML4+eCFP was replaced with that from pSG5PML-L, which encodes the C-terminus of PML isoform 3, to give rise to pMX+mycPML3+eCFP. To place PML and eCFP in frame, the C-terminus of PML3 was amplified by PCR using Phusion polymerase (New England Biolabs) and the primers 5'-aaaacgcgttgtggtgatcagcagctc-3' 5'aaaaccgcggagcgcgggctggtgggga-3', which removes the stop codon from PML3 and adds a SacII site. This PCR product was then used to replace the MluI-SacII fragment of pMX+mycPML3+eCFP to create pMX+mycPML3_eCFP.

The myc-tag was replaced with a FLAG-tag sequence by first subcloning the EcoRI-SphI fragment of pMX+mycPML3_eCFP into the same sites of pUC19 to create pUC19-(Nterm)mycPML. An oligomer encoding a 3xFLAG sequence (5'-atggactacaaagaccatgacggtgattataaagatcatgacatcgattacaaggatgacgatgacaac-3') was TOPO-cloned into pCR4Blunt-TOPO (Invitrogen) according to the manufacturer’s instructions to create pCR4Blunt-TOPO+3xFLAG. The BamHI-NcoI fragment from pCR4Blunt-TOPO+3xFLAG was used to replace the BamHI-NcoI fragment of pUC19+(Nterm)mycPML to create pUC19+(Nterm)3xFLAG_PML, the EcoRI-SphI fragment of which was then subcloned back into pMX+mycPML3_eCFP.

To enable interferon-inducibility of the integrated transgene, a quadruple repeat of the ISRE from *ISG15* flanked by XbaI restriction sites sites (5'-aaatctagacccgccccatgcctcgggaaagggaaaccgaaacgggaaagggaaaccgaaacgggaaagggaaaccgaaacgggaaagggaaaccgaaactgaagccaatctagaaaa-3') was inserted into the XbaI site of the 3'U3 element of pMX+3xFLAG_PML3_eCFP, giving rise to p3'415MX+3xFLAG_PML3_eCFP.

To create a form of PML that is resistant to silencing by shRNA produced in HA-shPML cells, a BamHI-fragment of pMX+3xFLAG_PML3_eCFP was subcloned into the vector, pAlter-1, and silent mutations were introduced using the Altered Sites Mutagenesis system (Promega) using the primer 5'-tgcatcacccaggggaaGgaCgcGgcGgtGAGTaaAaaGgccagcccagaggct-3' (with upper case letters representing introduced mutations) as per the manufacturer’s instructions. The BamHI fragment was then subcloned back into the parental vector to generate p3'415MX+3xFLAG_PML3R_eCFP.

To create a vector that encoded PML isoform 1, the C-terminus of PML-I was amplified by PCR (using the primers 5'- aaagcatgcagtgccccatctgc-3' and 5'-aaaaaaccgcgggctctgctgggaggccctctc-3') from a HEL-299 cDNA library and subcloned between the SacII and SbfI sites of p3'415MX+PML3R_eCFP to create p3'415MX+3xFLAG_PML1R_eCFP.

The PML phosphorylation knockout mutants were created using PCR mutagenesis with the following primers (with altered nucleotides indicated by capital letters):

S8A forward5'-ggagcctgcacccgcccgaGctccgaggccccagc-3'S8E forward5'-ggagcctgcacccgcTcgaGAAccgaggccccagcag-3'S8 reverse5'-atggcgttgtcatcgtcatccttg-3'T28;S36/38/40A forward5'-atgcctccccccgagGcGccctctgaaggccgccagcccGCTcccG-CGccGGCccctacagagcgagc-3'T28;S36/38/40E forward5'-atgcctccccccgagGAAccctctgaaggccgGcagcccGAAccc-GAAcccGAAcctacagagcgagcc-3'T28;S36/38/40 reverse5'-ggtgggctcctggggccgggcggg-3'S117A5'-agtctgcagcggcgcTtAGcggtgtaccggcaga-3'S117E forward5'-tctgcagcggcgcctCGAggtgtaccggcagat-3'S117E reverse5'-ctctcgaaaaagacgttatccagggcggg-3'S399/403;T409A forward5'-aaGgaCgcGgcGgtGGCTaaAaaGgccGCcccagaggctgcca-gcGctcccagggacccta-3'S399/403;T409E forward5'-aaGgaCgcGgcGgtGGAGaaAaaGgccGAAccTgaggctgcc-agcGAAcccagggaccctatt-3'S399/403;T409 reverse5'-cccctgggtgatgcaagagctgag-3'S480;T482;S493A forward5'-cagaagaggaagtgcGCGcagGcccagtgccccaggaaggtcatca-agatggagGctgaggaggggaagg-3'S480;T482;S493E forward5'-cagaagaggaagtgcGAGcagGAAcagtgcccTaggaaggtcatc-aagatggagGAAgaggaggggaaggag-3'S480;T482;S4935'-ggctgtcgttgtattggagacatc-3'S504/505A forward5'-ggcaaggttggctcgAGCcGccccggagcagccca-3'S504/505A reverse5'-tccttcccctcctcagactccatc-3'S504/505E forward5'-gcaaggttggctcggGAAGAGccggagcagcccagg-3'S505/505E reverse5'-ctccttcccctcctcagactccat-3'S518A forward5'-cagcacctccaaggcagtcGcaccTccTcacctggatggaccg-3'S518E forward5'-cagcacctccaaggcCgtGGAaccaccccacctgga-3'S518 reverse5'-ggcctgggctgctccgg-3'S527/530A5'-ctggatggaccgcctGCccccaggGCccccgtcataggaag-3'S527/530E forward5'-ctggatggaccgcctGAAccTaggGAAcccgtcataggaagt-3'S527/530E reverse5'-gtggggtggtgagactgccttggag-3'S560/561/562/565A forward5′-cgcgttgtggtgatcGCGGCcGcggaagacGcagatgccgaaaact-3′S560/561/562/565A reverse5'-ttcctctgcctccccggcgccact-3'S560/561/562/565E forward5'-ggaggcagaggaacgTgttgtggtgatcGAAGAAGAggaagacGA-Agatgccgaaaactcg-3'S560/561/562/565E reverse5'-ccggcgccactggccacgtggttg-3'S565A forward5'-agcagctcggaagacGcagatgccgaaaact-3'S565A reverse5'-gatcaccacaacgcgttcctctgc-3'S565E forward5'-gttgtggtgatcagcTCTtcggaagacGAagatgccgaaaactc-3'S565E reverse5'-gcgttcctctgcctccccggcgcc-3'V556/557/558A;I559S forward5'-aggcagaggaacgcgCtgCggCTaGcagcagctcggaaga-3'Δ476–490 forward5'-gaggcaaggttggctcgga-3'Δ476–490 reverse5'-ctgggctgtcgttgtattggaga-3'K65R5'-atgccaggcggaagcGCGCtgcccgaagctgctg-3'K160R forward5'-acaccagtggttcctACGTcaTgaAgcccggcccctagca-3'K160R reverse5'-gcctcgaagcacttggcgcag-3'K490R5'-cccaggaaggtcatcCGgatggagtctgagga-3'K616R forward5'-gttttctttgacctcCGgattgacaatgaaa-3'K616R reverse5'-cagaggtctgtcttctgcttggg-3'

In some cases, sequential rounds of PCR mutagenesis were required to introduce the desired mutations. All constructs were verified by sequencing.

Certain PML mutants were subcloned into pLNGY (a gift from Roger Everett) [12]. To enable rapid subcloning of the PML mutations from the p3'415MX+3xFLAG_PML1R_eCFP background, a PsiI-PsiI fragment (spanning the F1 origin of replication) from pLNGY was removed by restriction digest, and the vector was re-ligated to eliminate an AvrII restriction site. An AvrII-StuI region of the PML phosphorylation knockout mutants from p3'415MX+3xFLAG_PML1R_eCFP were subcloned into pLNGY(F1-).

### 2.3. Viruses

KOS [43] and Syn17+ [44] are wild type HSV-1 strains used in these studies. 7134 is an ICP0-null mutant in which the *ICP0* open reading frame of KOS is replaced by the *E. coli* *lac*Z gene [45]. *dl*1403/CMV*lacZ* is a Syn17+ ICP0-null virus containing a 2 kb deletion in both copies of the *ICP0* gene [46]. Both *dl*1403/CMV*lacZ* and another Syn17+ mutant, *in*1863 (which is otherwise similar to wild type Syn17+), encode *lacZ* under the control of the HCMV IE promoter inserted into the *tk* locus [13,47]. KOS, Syn17+, and *in*1863 viral stocks were prepared in Vero cells, and 7134 and dl1403/CMV*lacZ* were grown in U2OS cells; all viruses were titered as previously described [13,48,49].

Retroviruses were generated the Pantropic Retroviral Expression System (Clontech) as recommended by the manufacturer. Lentiviral stocks were generated essentially as for retroviral stocks except for the inclusion of the packaging vector, psPAX2 (Addgene plasmid 12260), and the use of HEK-293T cells for packaging [50].

### 2.4. PML Immunoprecipitation

HEL-299 cells were transduced with pseudotyped pMX+3xFLAG_PML3_eCFP such that the cells were 70% positive for PML3_eCFP. For uninfected samples, cells were plated in 10 100-mm dishes and grown to confluency. Twenty-two µg of anti-FLAG (M2) were added to 165 µL Dynabeads protein G (Invitrogen), which were washed and prepared according to the manufacturer’s instructions, and allowed to incubate together at 4 °C overnight. The cells from each plate were washed with 1 mL PBS containing protease inhibitors (1 µg/mL aprotinin, 1 µg/mL leupeptin, 10 mM phenylmethanesulfonylfluoride, 1 mM Na_3_VO_4_, 1x Complete protease cocktail inhibitor (Roche)), and scraped into PBS, centrifuged to pellet cells, and resuspended in 200 µL lysis buffer (4% SDS, 10 mM dithiothreitol, 300 mM NaCl, 100 mM HEPES (pH 7.5)) [51] containing inhibitors. The samples were solubilized by incubation at 100 °C for 5 minutes, vortexed, and sonicated at 100 W for 1 minute using a cup sonicator. The samples were then combined and diluted with 13 mL of diluent buffer (1.7% Thesit, 150 mM NaCl, 50 mM Hepes (pH 7.5)) containing the protease inhibitors. The anti-FLAG-conjugated Dynabeads were added to the lysate and incubated at 4 °C overnight. The next day, the beads were precipitated using a magnet and washed with 2 mL of the diluent buffer (containing inhibitors) 4 times. After the final wash, the beads were resuspended in 50 µL of Laemmli buffer [52], boiled for 5 minutes, and resolved on 4%–20% Tris-glycine SDS-PAGE gels. The gels were stained with Coomassie blue, thoroughly destained, and the desired bands were excised and washed with a 50% acetonitrile/water solution.

PML from infected cells was prepared essentially as above except that cells were pretreated with 10 µM MG132 for 1 h, infected with KOS at an estimated 5 PFU/cell in the presence of MG132, and cells were collected at 5 hpi.

### 2.5. Mass Spectrometry (MS)

To maximize sequence coverage of PML from both uninfected and infected cells, excised gel bands were subjected to digestion with trypsin, chymotrypsin and elastase followed by mass spectrometry. Peptide sequence analysis of each digestion mixture was performed by microcapillary reversed-phase high-performance liquid chromatography coupled with nanoelectrospray tandem mass spectrometry on an LTQ-Orbitrap Velos mass spectrometer (ThermoFisher Scientific, San Jose, CA). The Orbitrap repetitively surveyed an *m*/*z* range from 395 to 1600, while data-dependent MS/MS spectra on the twenty most abundant ions in each survey scan were acquired in the linear ion trap. MS/MS spectra were acquired with relative collision energy of 30%, 2.5-Da isolation width, and recurring ions dynamically excluded for 60 s. Preliminary sequencing of peptides was facilitated with the SEQUEST algorithm [53] with a 30 ppm mass tolerance against the Uniprot Knowledgebase human reference proteome supplemented with a database of common laboratory contaminants, concatenated to a reverse decoy database. Using a custom version of Proteomics Browser Suite (PBS v.2.7, ThermoFisher Scientific), peptide-spectrum matches (PSMs) were accepted with mass error <2.5 ppm and score thresholds to attain an estimated false discovery rate of ~1%. Data-sets for all digest results were combined in silico, culled of minor contaminant PSMs, and re-searched with SEQUEST against the PML sequence without taking into account enzyme specificity and with differential modifications of phosphorylated tyrosine, serine, and threonine residues. The discovery of phosphopeptides and subsequent manual confirmation of their MS/MS spectra were facilitated by in-house versions of programs MuQuest, GraphMod, and FuzzyIons (Proteomics Browser Suite, ThermoFisher Scientific.)

### 2.6. Western Blots

To examine the ability of ICP0 to induce degradation of PML or its mutant forms, HEp-2 cells were plated at 1 × 10^5^ cells per well of a 24-well plate. The cells were transfected 24 h later with either 100 ng of p3'415MX+3xFLAG_PML1R_eCFP or one of the PML phosphorylation mutants along with 1 µg of pcDNA3.1, pcDNA3.1+n212, or pcDNA+ICP0 using Lipofectamine 2000 (Invitrogen) as per the manufacturer’s recommendation. At 24 hours post transfection, the cells were washed once with PBS and lysed into 50 µL of boiling Laemmli buffer containing 1 µg/mL aprotinin, 1 µg/mL leupeptin, 1 mM PMSF, 10 mM sodium vanadate, 50 mM sodium fluoride, and 20 mM N-ethylmaleimide. One-fifth of each sample was resolved on 4%–12% Bis-tris polyacrylamide gels, transferred to nitrocellulose, blocked at room temperature for 1 h with 2% nonfat dry milk in Tris-buffered saline with 0.1% Tween 20 (TBS-T). The blots were probed either overnight at 4 °C or for 2 hours at room temperature with primary antibodies. Primary antibodies used included those directed against FLAG (M2, Sigma Aldrich) or β-actin ((I-19)-R, Santa Cruz Biotechnology). Antibodies were diluted in 2% non-fat dry milk/TBS-T. Membranes were then washed three times with TBS-T and probed at room temperature with goat-anti-mouse IgG, or goat-anti-rabbit IgG conjugated to HRP (Jackson Immunoresearch). Membranes were again washed with TBS-T and developed with chemiluminescent substrate (Femto ECL, Pierce Laboratories). Chemiluminescence was detected using an Image Station 4000R (Kodak) and Carestream Molecular Imaging software. Images were assembled using Adobe Photoshop and Illustrator (Adobe Systems), and band intensities were measured by densitometry analyses using ImageJ.

To examine SUMOylation levels of PML, 1 × 10^5^ HEp-2-shPML cells were plated per well in 24-well plates. The next day, the cells were transfected with 100 ng of p3'415MX+3xFLAG_PML1R_eCFP or one of the PML phosphorylation mutants along with 900 ng of pGEM-3 using Lipofectamine 2000 according to the manufacturer’s instructions. At 24 h post transfection, the cells were lysed as above. One fifth of each sample was resolved, transferred, and probed as above except that proteins were resolved using 6% Tris-glycine polyacrylamide gels. Images were assembled using Adobe Photoshop and Illustrator (Adobe Systems) and band intensities were measured by densitometry analyses using ImageJ (National Institutes of Health).

### 2.7. Immunofluorescence/Fluorescence Microscopy

To examine the ability of exogenous PML to recruit Sp100 and Daxx to ND10s, A549-shPML or HA-shPML cells were transduced with retroviral vectors encoding 3xFLAG_PML1R_eCFP or one of the PML-I phosphorylation site mutants generally as described for the creation of depleted cells. These cells, as well as A549 and HA-shNeg cells, were plated on collagen coated coverslips and the next day were washed once with PBS, fixed for 5 minutes with 5% formaldehyde in PBS at room temperature, washed three times with PBS, permeabilized at 4 °C for 15 minutes with 0.5% NP-40 in PBS, and washed an additional three times with PBS. Coverslips were the probed for 30 minutes at 37 °C with antibodies against Sp100 (mAb1380, Millipore) and Daxx (S-20, Santa Cruz Biotechnology) diluted in 1% FCS, 1% BSA, 0.05% Tween-20 in PBS; A549 and HA-shNeg cells were probed with antibodies against PML (A301-167A, Bethyl Laboratories) and Sp100. Cells were washed three times with PBS and stained for 30 minutes at 37 °C with donkey-anti-mouse IgG Dylight 594 and cow-anti-goat IgG Dylight 488 diluted in the same buffer; A549 and HA-shNeg cells were stained with donkey-anti-rabbit IgG Dylight 594 and donkey-anti-mouse IgG Dylight 488. Coverslips were washed three times with PBS, air dried, and mounted onto glass slides using ProLong antifade (Invitrogen). Proteins were viewed by confocal fluorescent microscopy (Nikon) and captured with a digital camera (Photometrics). Images were assembled using Adobe Photoshop and Illustrator (Adobe Systems). ND10 reformation in HA-shPML was examined in a similar manner, except that the antibodies used were against Sp100 and Daxx (sc-16328, Santa Cruz Biotechnology and mAB 5.14 [54], respectively). At least 20–60 cells were counted for the ND10 reformation studies with PML-I or each of its mutant forms.

To examine colocalization between PML-I and ICP0, A549-shPML, HA-shPML cells, and their derivatives were transduced as above. Two days post transduction, the cells, as well as A549 and HA-shNeg cells, were infected with KOS at an estimated 2.5 PFU/cell. To establish a time line for colocalization, an initial set of cells was fixed at 1, 2, 3, 4, 5, and 6 hpi as described above and all subsequent studies were examined at 2 hpi. The transduced and infected cells were probed first with antibodies against ICP0 (H11060, Santa Cruz Biotechnology) and ICP4 (EastCoastBio) diluted in 5% rabbit serum in PBS and then probed with goat-anti-mouse IgG1 Dylight 594 and goat-anti-mouse IgG2b Dylight 488 as listed above for Sp100 and Daxx staining. A549 and HA-shNeg cells were stained first for endogenous PML (A301-167A) and ICP0 (H11060) and then with donkey-anti-rabbit IgG Dylight 594 and goat-anti-mouse IgG2b Dylight 488. At least 50 cells were examined for ICP0’s colocalization with PML-I or each of its mutant forms.

To examine the recruitment of PML-I or the PML-I mutants to incoming viral genomes, HA-shPML cells were transduced described above. Three days post transduction, the medium was removed, and cells were infected with dl1403/CMVlacZ at an estimated MOI of 0.1. At 1 hpi, the cells were overlaid with growth medium containing 0.5% methylcellulose. At 24 hpi, the medium was removed, the cells were washed with PBS, and the cells fixed and permeabilized by incubation at −20 °C in cold 20% acetone diluted in methanol for 15 min. Samples were then washed and stained with an antibody against ICP4 as described above. HA-shNeg cells were also infected as described and were additionally stained with antibody against PML as described for the ICP0 colocalization studies. At least 20 cells were examined for the recruitment activity of PML-I or each PML-I mutant to incoming viral genomes.

## 3. Results and Discussion

### 3.1. Identification of Phosphorylated Residues of PML in Uninfected and HSV-1 Infected Cells

While PML is known to be phosphorylated on a number of sites when this project began, a full mapping of phosphorylated sites on PML had not been performed. Additionally, PML phosphorylation during infection had not been examined. To address these questions, we created in human embryonic lung (HEL-299) cell line that exogenously expresses a FLAG- and CFP-tagged form of PML-III. PML was immunoprecipitated from cells mock-infected or infected with HSV-1 for 6 h, and PML bands purified by from SDS-PAGE were subjected digestion with trypsin followed by tandem mass spectrometry. As detailed in Table 1, we identified 19 phosphorylated serines and threonines in uninfected cells and 11 phosphorylated sites in infected cells, though we noted that there were significant gaps in the coverage of our scans, particularly in the N-terminus of PML-III. Of the sites identified, those located within regions shared among the different isoforms (from residues 1–575) were either previously identified or reported in subsequent studies; we did, however, find several novel sites within a C-terminal region specific to PML-III (each indicated by an asterisk). Of the sites identified, we found that S504 was phosphorylated only in uninfected cells, while S565 was only detected as phosphorylated in infected cells. Thus, we have identified a number of novel specific sites of phosphorylation on PML-III and that there appears to be changes in the phosphorylation status of PML upon infection.

**Table 1:** Sites of Phosphorylation on PML-III.

Uninfected		HSV-1 Infected	
	Coverage gaps		Coverage gaps
S399	1–44	S403	1–7
S403	57–86	T409	18–44
T409	147–149	S505	57–97
S480	212–216	S518	132–153
T482		S527	206–216
S493		S565	336–337
S504		T594*	395–399
S505		S598*	478–486
S518		S603*	
S527		S613*	
T594*		S637*	
S598*			
S603*			
S608*			
S613*			
S616*			
S619*			
T620*			
S637*			

### 3.2. Phosphorylation at Sites near the SIM Alter ND10 Morphology and Influences Sp100 and Daxx Recruitment to ND10s

To examine the effect that phosphorylation at these sites has on PML, we made a series of retroviral constructs encoding FLAG- and CFP-tagged phosphorylation knockout and mimetic mutants, changing residues to alanine or glutamic acid, respectively. We additionally made forms of PML bearing mutations in the SIM (V556/557/558A;I559S) [6], the major SUMOylation acceptor sites (K65R, K160R, K490R, or K65/160/490R) [21], and the nuclear localization signal (NLS) (Δ476–490) [55]. During the course of this work, it was shown that PML-I is the most widely expressed of the PML isoforms [56] and, unlike PML-III, has antiviral activity toward certain HSV-1 mutants [12]. Thus, these phosphorylation mutants were made in a PML-I background. Because exogenous PML forms heterodimers with endogenous PML, which would complicate the interpretation our data, we made cell lines that express shRNAs directed against PML and introduced the mutant forms of PML-I (which contained additional silent mutations to enable shRNA-resistance) into them. Initially, we chose A549 cells, a human lung epithelial carcinoma cell line, because they are immortalized and have a functional interferon-based antiviral response to a number of viruses [57,58,59,60,61,62,63,64,65,66], and we found them to be amenable to depletion of PML using shRNAs.

**Figure 2:**
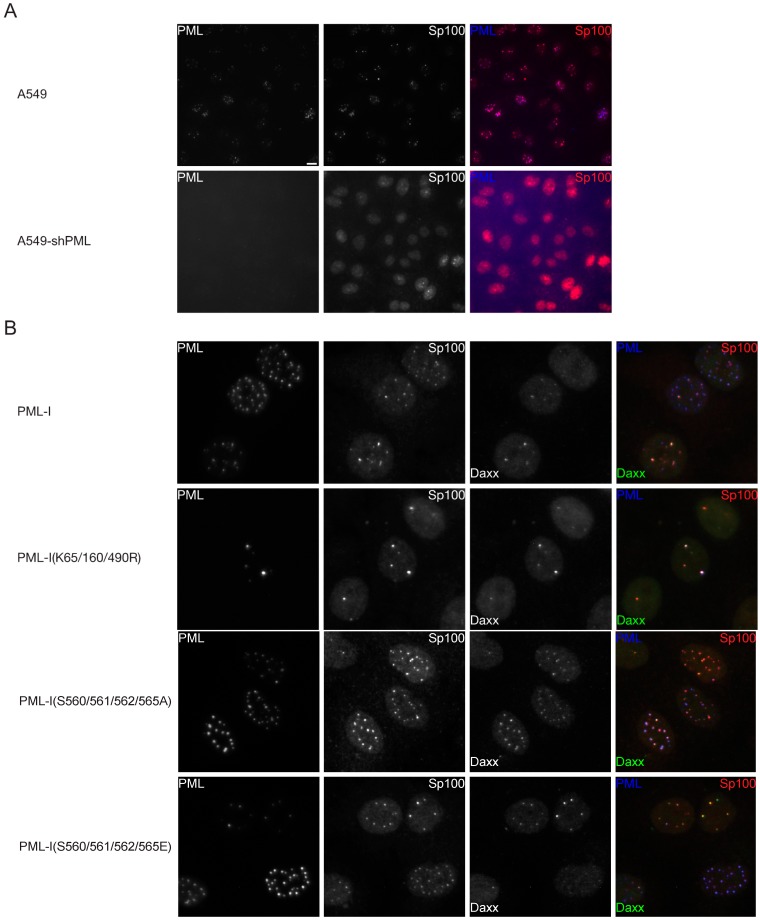
Phosphorylation sites near the SIM control ND10 size and number. Recruitment of Sp100 and Daxx to PML-I and PML-I phosphorylation mutants in PML-depleted A594 cells. (**A**) A549 or A549 cells that express an shRNA that targets PML (A549-shPML) and stained for PML and Sp100 by immunofluorescence. (**B**) A549-shPMLs were transduced with FLAG-, eCFP-tagged PML-I, a SUMOylation-deficient mutant, or one of two phosphorylation mutant form of PML-I. Sp100 and Daxx were detected by immunofluorescence, and exogenous PML was detected by autofluorescence. Scale bar = 10 μm.

We first examined the ability of exogenous PML to form ND10s and to recruit the major ND10 constituents, Sp100 and Daxx. As expected, cells transduced with the shRNA targeting PML were largely devoid of PML and Sp100 was largely diffuse in the nucleoplasm in contrast to the parental A549s (Figure 2A). In cells transduced with the PML-I-expressing retrovirus, however, ND10s were clearly present along with, to a degree, colocalized Sp100 and Daxx (Figure 2B and Figure S1). An initial examination of the mutants failed to reveal large differences in ND10 size or number for most of the PML phosphorylation mutants (Figure S1), with the exception being those bearing mutations near the SIM, S560/561/562/565A and –E, in which case the ND10s were larger; likewise, none of the mutants completely failed to recruit Sp100 or Daxx, though, again, those mutated near the SIM appeared different in that they were able to recruit both Sp100 and Daxx much more efficiently. These results were also true when PML expression was restored in a HepaRG-based PML-depleted cell line (Figure S2), though the PML-I(K65/160/490R) SUMOylation-deficient mutant failed to recruit Sp100 and Daxx in these cells.

**Figure 3:**
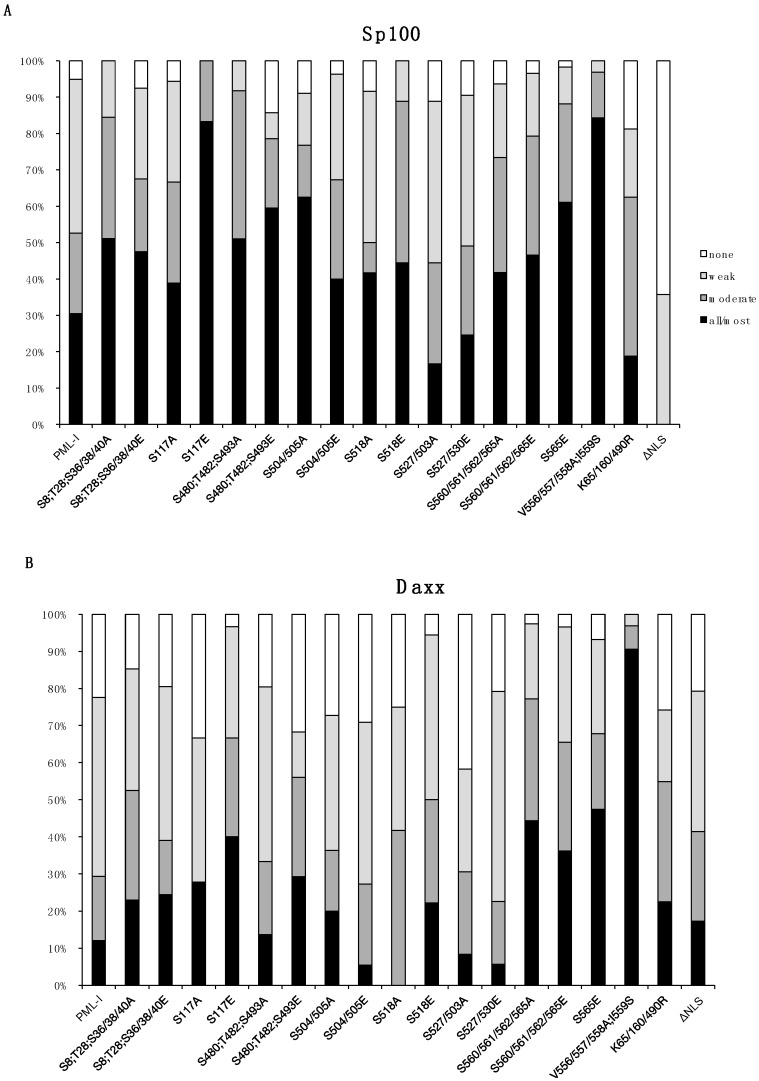
Quantification of Sp100 and Daxx recruitment to PML in PML-depleted A549 cells that express wild type and mutant forms of PML-I. A549-shPML cells transduced with CFP-tagged PML-I or each PML-I mutant were scored on a four-point scale based on how well they recruited Sp100 (A) or Daxx (B), with either none (none), less than 50% (weak), between 50%–90% (moderate), or >90% (all/most) of the protein being localized at ND10s, as judged using fluorescence microscopy.

A more detailed examination of ND10s revealed subtle differences among the PML-phosphorylation mutants in their ability to recruit Sp100 and Daxx (Figure 3). Notably, the S117E and S518E mutants were much more likely to recruit Sp100 and somewhat better at recruiting Daxx, and specifically for S117E, Sp100 colocalized with it in all cells that were examined. Of the remaining mutants, we also found that S8/T28/S36/38/40A, S480/T482/S493A, S480/T482/S493E, and S504/505A mutants were marginally better at recruiting Sp100. These data suggest that phosphorylation near the SIM, and potentially S117, has an effect on Sp100 and Daxx recruitment and that phosphorylation at several other sites on PML appear to subtly influence ND10 member recruitment.

### 3.3. Phosphorylation does not Largely Impact SUMOylation Levels

**Figure 4:**
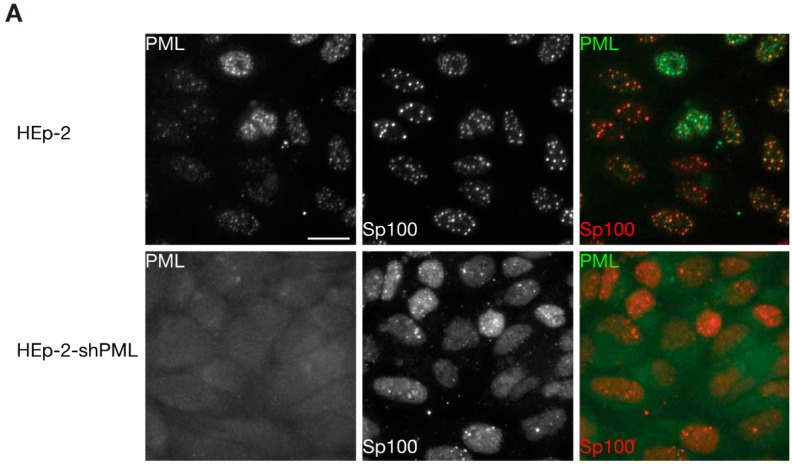

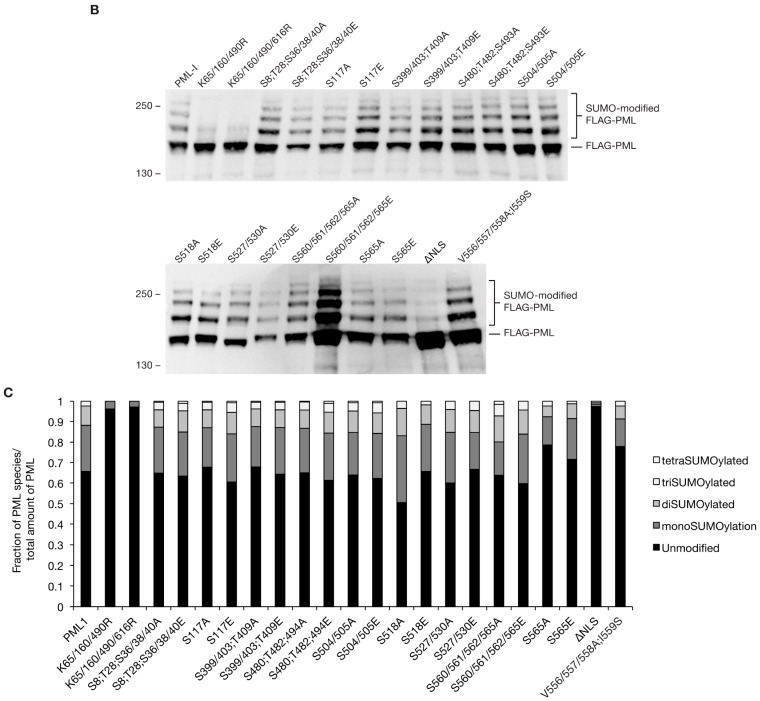
Mutation of PML phosphorylation sites results in minor changes in the SUMOylation state of PML-I. (**A**) HEp-2 cells and HEp-2 cells depleted of PML and stained with antibodies to detect PML and Sp100 by immunofluorescence. Scale bar = 10 μm. (**B**) HEp-2-shPML cells were transfected with 900 ng of pGEM-3 (as carrier DNA) and 100 ng of a plasmid encoding FLAG-, eCFP-tagged PML-I or a mutant of PML-I. The next day, the lysates of the cells were prepared, resolved by SDS-PAGE, and analyzed by western blot with an anti-FLAG antibody. (**C**) A densitometry analysis was performed for the western blot from B. Chart shows the relative fraction that each band represents of the total amount of PML present.

As previous reports suggested interplay between phosphorylation and SUMOylation and current evidence suggest that PML acts as an E3 SUMO ligase, perhaps on itself, we decided to examine the SUMOylation state of the PML phosphorylation mutants. HEp-2 cells, which have previously been used to examine PML post-translational modifications in the context of an HSV-1 infection [39], were depleted for PML (HEp-2-shPML) (Figure 4A), allowing us to examine the E3 ligase activity of PML without endogenous PML affecting the interpretation of our results. The cells were transfected with the S8;T28;S36/38/40A, S8;T28;S36/38/40E, S117A, S117E, S399/403;T409A, S399/403;T409E, S480;T482;S493A, S480; T482;S493E, S504/505A, S504/505E, S518A, S518E, S527/530A, S527/530E, S560/561/562/565A, S560/561/562/565E, S565A, and S565E phosphorylation mutants, the K65/160/490R and K65/160/490/616R SUMOylation-deficient mutants, the NLS mutant, or the SIM mutant, and the next day cell lysates were prepared and examined by western blot. While FLAG-, eCFP-tagged wild type PML-I exhibited the laddering typical of multiply SUMOylated proteins (Figure 4B), both the K65/160/490R and K65/160/490/616R mutant forms of PML-I were largely devoid of SUMO modification. These bands likely represent SUMOylation of minor, non-preferred or non-canonical sites [22,23]. Likewise, the NLS-deletion mutant showed decreased SUMOylation while the SIM-mutant was slightly less SUMOylated than wild type. Of the phosphorylation mutants, while no gross differences in SUMOylation patterns were obvious, densitometric analysis (Figure 4C) showed that the S518A mutant was more highly SUMOylated while the S565A mutant was slightly less so, resembling the SIM-mutant. These results indicate that the phosphorylation sites examined do not greatly affect SUMOylation levels of PML in resting cells.

### 3.4. Phosphorylation is not Required for the Colocalization of PML-I and ICP0

**Figure 5:**
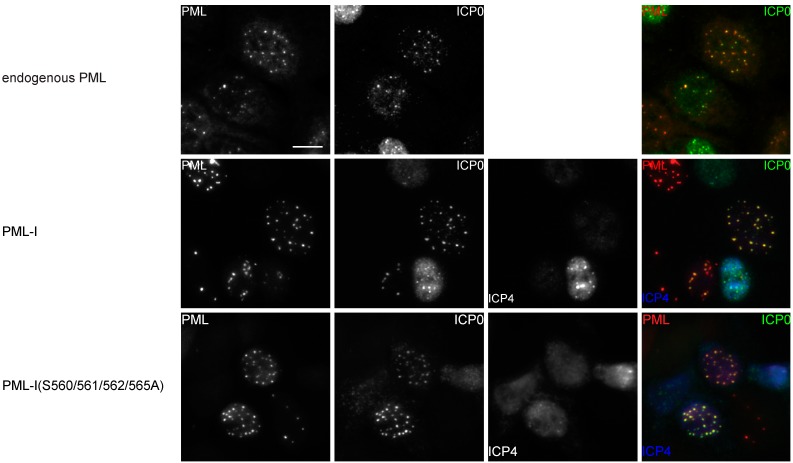

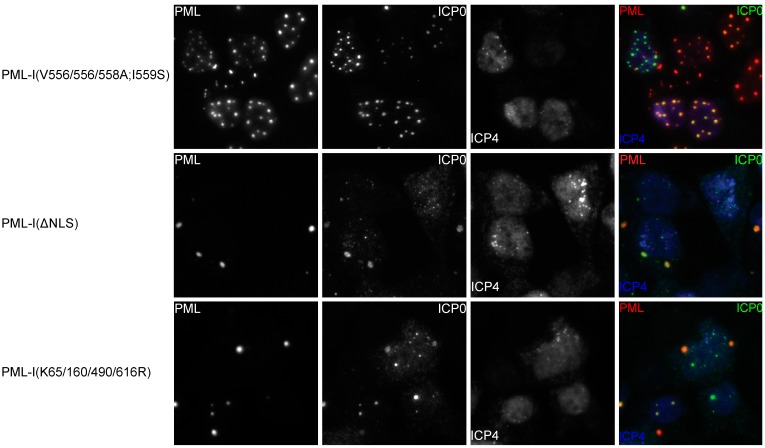
Mutation of PML phosphorylation sites does not affect colocalization of PML-I and ICP0 during HSV-1 infection. HA-shPML cells transduced with FLAG-, CFP-tagged PML-I or each PML-I mutant were infected with HSV-1 at 2 PFU/cell. At 2 hpi, the cells were fixed and stained with antibodies against ICP0 and ICP4. PML is shown as red, ICP0 as green, and ICP4 as blue in the merged image. Scale bar = 10 μm.

The ability of ICP0 to interact with PML has been reported to be facilitated by PML SUMOylation [38,39]. Forms of ICP0 that do not colocalize with PML either fail to or are inefficient at inducing the degradation of PML [67]. As phosphorylation of PML can influence PML SUMOylation and potentially block or create a binding site for ICP0, we wished to determine whether PML phosphorylation affected PML:ICP0 colocalization. While ICP0 induces the proteasomal-dependent degradation of PML, the use of proteasome inhibitors has been reported to change ND10 composition. To determine a time point at which ICP0 levels were high enough for detection but had not yet induced substantial ND10 disassociation, PML-I-transduced HA-shPML cells were infected with HSV-1 and processed for immunofluorescence assays at 1, 2, 3, 4, 5, and 6 hpi (data not shown). We found that 2 hpi was an ideal time at which to examine PML:ICP0 colocalization. HA-shPML cells were transduced with retroviral vectors encoding PML-I or one of the PML-I phosphorylation knockout mutants and two days later infected with HSV-1 at 2.5 PFU per cell to ensure at least 99% of all cells were infected. At 2 hpi, cells were fixed and processed for ICP0 immunofluorescence staining, with PML detected by autofluorescence. As expected, we found that in infected cells expressing PML-I, ICP0 was present at all ND10s (Figure 5 and Figure S3). When we examined infected cells transduced with the PML-I phosphorylation mutants, we again found that ICP0 was present at a majority of PML bodies. Likewise, we found colocalization between PML and ICP0 in cells expressing only PML mutated at the major SUMOylation sites, though as noted in Figure 4, this mutant retained a small amount of SUMOylation. To confirm that ICP0 was at ND10s due to the presence of PML and not another ND10 constituent (such as SUMOylated Sp100), we also tested the ability of a PML NLS-deletion mutant to retain ICP0 in the cytoplasm. PML-I(ΔNLS) was, as expected, restricted to the cytoplasm, where it formed 2–3 puncta per cell. In cells transduced with this construct, ICP0 was strongly retained in the cytoplasm. We also examined colocalization between ICP0 and PML-I, the PML-I phosphorylation knockout, PML-I phosphorylation mimetic, SIM, NLS, and K65/160/490/616R mutants in the A549-shPML cells (Figure S4) and found that they recapitulated our results in the HA-shPMLs cells. These assays indicate that either none of the phosphorylation sites we examined greatly influence the ability of ICP0 to localize with PML-I or that there appears to be multiple sites on or regions in PML to facilitate this process.

### 3.5. PML Phosphorylation has Minor Effects on ICP0-Induced Degradation

**Figure 6:**
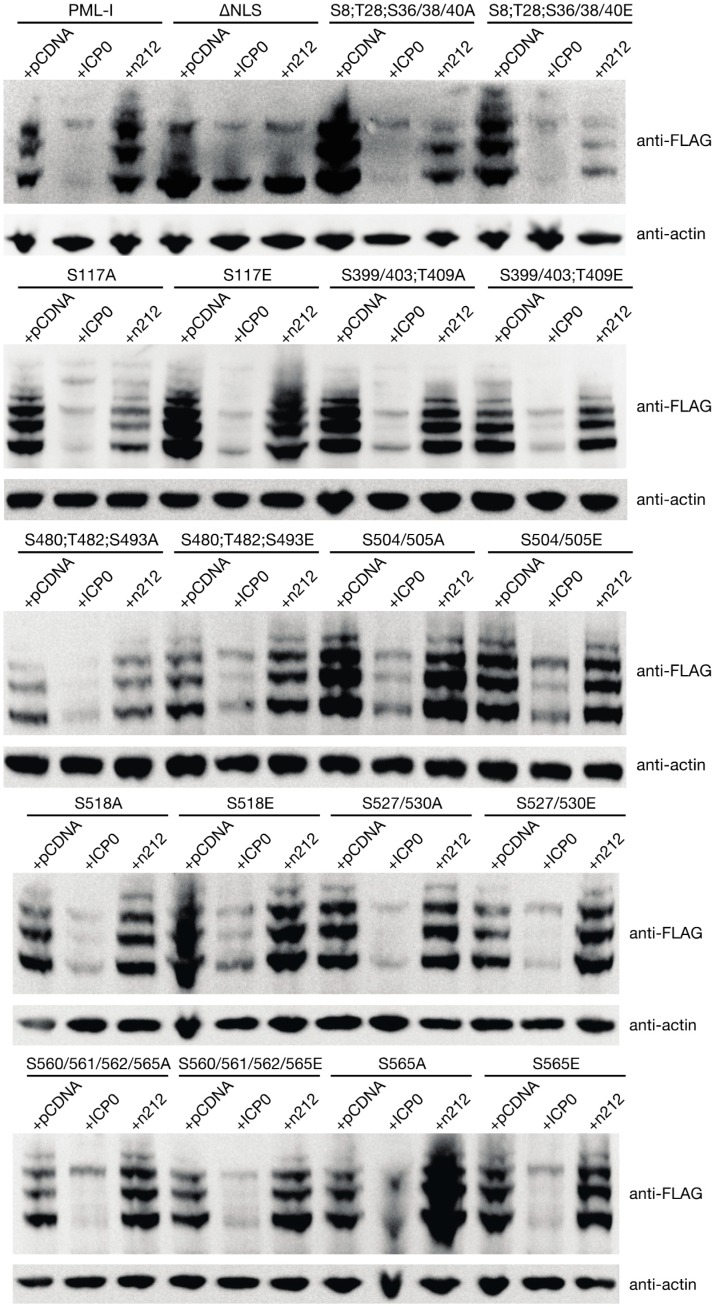
ICP0 induces degradation of PML-I regardless of mutated PML phosphorylation sites. HEp-2 were transfected with 900 ng of an empty vector (pGEM-3), a vector encoding ICP0, or a vector encoding the ICP0 mutant n212 and 100 ng of a plasmid encoding FLAG-, eCFP-tagged PML-I or a PML-I mutant. Twenty-four hours later, cells were lysed, resolved by SDS-PAGE, and analyzed by western blot with an anti-FLAG or anti-β-actin antibody.

Phosphorylation of PML has been shown to contribute to the stability through a number of mechanisms. To more directly assay whether any of the phosphorylation sites influence the stability of PML in the presence of ICP0, we cotransfected the retroviral constructs that express PML-I or its mutant forms along with either an empty control vector, a vector encoding ICP0, or a vector encoding the n212 truncation mutant of ICP0 (which is incapable of inducing PML degradation) into HEp-2 cells, which have previously been used in assays examining ICP0-induced PML degradation [39]. Twenty-four h later, the levels of exogenous PML were examined by western blot. As expected, expression of ICP0 lead to the near total loss of PML while expression of the n212 mutant form of ICP0 largely did not (Figure 6). Interestingly, while the NLS mutant and ICP0 colocalize (Figure 5 and Figure S4), ICP0 was unable to induce its degradation. Examination of the phosphorylation mutants revealed that ICP0 was capable of reducing the levels all of these mutants; however, we noted that levels of the S480;T482;S493E, S504/505A, S504/505E, and S518E mutants, while lower in the presence of ICP0, were not diminished to the same degree as wild type PML-I. These results indicate that phosphorylation may play a role in determining PML stability in the presence of ICP0.

### 3.6. Mutation of the Phosphoacceptor Sites in the Phospho-SIM of PML-I Prevents its Recruitment to Incoming Viral Genomes

As viral DNA is injected into the nucleus, it is recognized as either foreign or damaged DNA and as such, the cell mounts a response involving DNA damage factors, components of the SUMOylation machinery, and ND10 members. In the case of ND10s, preexisting ND10s breakdown and recoalesce around viral genomes; preventing these cellular factors from accumulating around viral DNA correlates with a loss in their antiviral activity towards HSV-1. In the case of PML, it has been shown that both SUMOylation of PML and the integrity of its SIM are required for relocalization at incoming genomes. Consequently, we wished to determine whether phosphorylation of PML played a role in this process.

**Figure 7:**
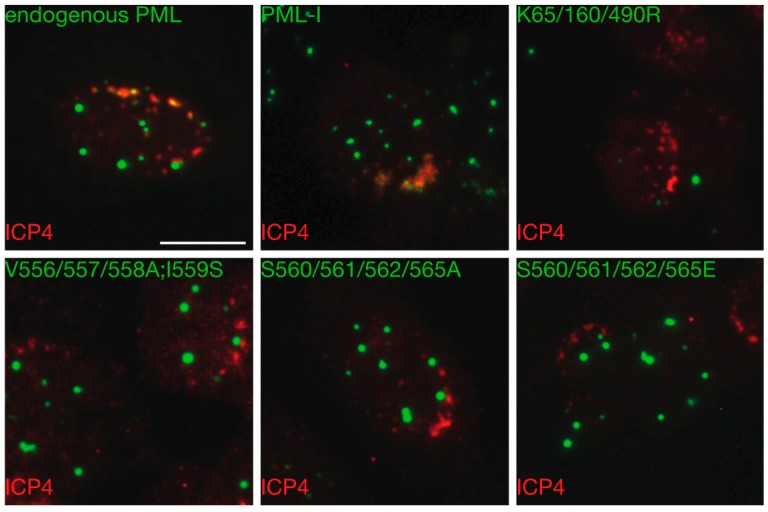
Mutation of phosphorylation sites near the SIM compromises recruitment of PML-I to incoming HSV-1 genomes. HA-shPML cells transduced with FLAG-, eCFP-tagged PML-I or a PML-I mutant were infected with an ICP0-null virus at 0.1 PFU/cell. At 24 hpi, the cells were fixed and stained with antibody against ICP4 as a marker of viral DNA. Scale bar = 10 μm.

The recruitment of intrinsic antiviral effectors to incoming viral genomes can most easily be seen at the edge of spreading plaques on monolayers infected at low MOIs with ICP0-null mutant viruses. Cells at the plaque edge experience a directional infection, with viral capsids primarily docking and injecting the viral DNA on one side of the cell. This can be visualized by staining for the major viral transactivator, ICP4, which binds to viral DNA. Therefore, HA-shPML cells were transduced with the various PML-I retroviral constructs. Four days later, they were infected with an ICP0-null virus at a low multiplicity of infection, and overlaid with methylcellulose to restrict viral spread. The next day, the cells were fixed and stained for ICP4, with PML detected by autofluorescence. As expected, along the edges of plaques we observed cells in which ICP4 was found as a front along one side of the nuclear envelope (Figure 7 and Figure S5). In cells transduced with PML-I, we found a strong recruitment to ICP4 foci whereas PML-I(V556/557/558A;I559S), in which the SIM is inactivated or PML-I(K65/160/490R), in which the major SUMOylation acceptor sites are mutated, failed to show any relocalization to ICP4 fronts (Figure 7). Of the various PML phosphorylation mutants, we found that only those that were mutated at sites in the phosphorylation region adjacent to the SIM failed to relocalize to incoming genomes. In all cases observed, both the S560/561/562/565A and S560/561/562/565E mutants failed to colocalize with ICP4, indicating that phosphorylation of the SIM may be required for PML’s antiviral activity. Indeed, it has previously been shown that mutation of these serines to alanine decreases the ability of PML to interact with SUMOylated proteins in yeast two-hybrid assays [68] and in bioluminescence resonance energy transfer assays [69]. Thus, we have found that of the phosphorylation sites observed in PML, only mutation of those in the phosphoSIM appear to compromise the ability of PML to be recruited to incoming viral genomes.

## 4. Conclusions 

PML is constitutively expressed and as such, can respond immediately to cellular stimuli; however, to do so, it must be regulated in a dynamic manner. One method of quickly controlling proteins includes altering their post-translational modification state. PML is known to be extensively modified and, in some instances, its post-translational state is changed in response to various stimuli when functioning in particular cellular pathways. For instance, phosphorylation of PML has been tied to its role in cell cycle control [31], differentiation [70], and the DNA damage response [71]. While PML has been demonstrated to have antiviral activity towards a number of viruses, the role of phosphorylation in this response has received little attention.

When this work was began, only a few studies had been performed that mapped phosphorylation on PML. We and others have since made use of advances in mass spectrometry technology to perform precise mapping studies of PML phosphorylation (Figure 1) [29,32,40,72,73,74]. We are the first group to examine PML phosphorylation during viral infection. Notably, we have mapped a cluster of sites that match the S/T-Q phosphoinositide 3-kinase-related kinase (PI3KK) consensus motif [75]. In addition to these potential PI3KK acceptor sites, we found that serine 504 was phosphorylated only in uninfected cells and serine 565 was so only in infected cells. Phosphorylation of S565 by CK2 has previously been shown to promote PML polyubiquitination as well as increase the affinity of the SIM for SUMO [31,69]; however, as we have previously noted, the use of pharmacological inhibitors of CK2 does not prevent the degradation of PML by ICP0 [76], and we failed to observe that mutation of S565 alone increases the stability of the PML-I in the presence of ICP0.

A major function of PML is the nucleation and recruitment of other ND10 member proteins. Two proteins of note, particularly in terms of the antiviral effect mediated by ND10s, are Sp100 and Daxx, especially as these proteins cooperate to limit HSV-1 replication [42]. When we examined exogenous PML-I expressed in PML depleted cells, we found that only a small proportion of the reformed ND10s strongly recruited Sp100 and Daxx (Figure 2 and Figure 3). This is in agreement with previous results showing that expression of individual isoforms of PML failed to fully restore recruitment of Sp100 and Daxx. The PML-I phosphorylation mutants generally behaved like wild type PML-I when it came to the recruitment of Sp100, although the ND10s formed by the S117E, S480;T482;S493E, and S504/505A mutants appeared to have a higher degree of Sp100 recruitment (Figure 3A). For the most part, Daxx recruitment followed a similar trend to that of Sp100, though overall it appeared much more likely to be nucleoplasmic (Figure 2 and Figure 3B). The most notable exceptions were the S560/561/562/565A and S560/561/562/565E mutants, both of which were more likely to have intense Daxx staining at the reformed ND10s and Sp100 was more likely to localize with PML. Phosphorylation of PML might influence Sp100’s ability to either localize to ND10s or interact with an adaptor protein and results in an indirect effect on PML:Sp100 colocalization. Daxx, however, directly interacts with PML in a manner that requires both SUMOylation of PML and a SIM present in Daxx [6,25]. In other studies involving depletion and restoration of PML expression, it was noted that either PML-VI, which lacks the SIM, or mutants of the other isoforms in which the PML SIM was mutated recruited Sp100 and Daxx more readily than wild type versions of PML-I though –V. Thus, it seems likely that the PML SIM is required for interaction with another protein that negatively regulates the interaction between Sp100 and Daxx with PML-I; however, the only proteins known to require the PML SIM for interacting with PML are subunits of the proteasome [77].

Early during infection, ICP0 colocalizes with PML and induces the proteasomal degradation of PML as well as the loss of certain forms of Sp100 [37]. Though not strictly required for the ability of ICP0 to induce the loss of PML, forms that fail to localize to ND10s are inefficiently degraded [67]. We found that mutation of phosphorylation sites in PML-I did not noticeably affect the colocalization between PML and ICP0 (Figure 5). Recent work has shown SUMOylation of PML-II, -III, -IV, -V, and –VI is necessary for their interaction with ICP0, while a region in the C-terminus unique to PML-I has the ability to interact with ICP0 in a SUMOylation-independent manner [38]. Little is known about this region other than it is predicted to have an exonuclease-III domain and that it is necessary for nucleolar-localization of PML-I in senescent cells or those that have been induced to have double stranded DNA breaks [78].

Entry of viral DNA into the nucleus triggers the deposition of DNA damage response factors, SUMOylation machinery, and ND10 components at or near the viral DNA [16,79,80]. While the specific host cell activator of these factors in response to viral infection has not been identified, it is notable that many of the same factors assemble around sites of DNA damage [81]. Recruitment of PML to incoming viral genomes, which is essential for its antiviral function, requires the RING-finger, B-box 1, coiled-coil, SIM, and SUMOylation on either K160, K490, or both [36]. In the case of DNA damage, ND10s undergo an ATM-, CHK2-, and ATR-dependent fragmentation where portions of preexisting ND10s bud off and move to sites of damage, indicating that phosphorylation of PML may play a role in this process [82]. It is unknown whether this phosphorylation-dependent breakdown of ND10s is also required for recruitment to viral DNA, though the deposition of ND10 components near viral DNA does first require the exchange between ND10s and the nucleoplasm [16].

Of the phosphorylation mutants examined, we found that only sites near the SIM detectably influence recruitment of PML to incoming viral genomes (Figure 7). Phosphorylation of the phospho-SIM motifs of Daxx and PIAS1 have been shown to affect the ability of the SIM-domain containing protein to interact with SUMO, and in the case of Daxx, determining SUMO paralog preference [83], although this was not the case for PML [69]. Curiously, mutation of the serines of the phospho-SIM motif to either alanine, which would block phosphorylation, or glutamic acid, which should mimic phosphorylation, both lead to the same phenotype. In this instance, it might be that the reversible phosphorylation of the phospho-SIM is necessary for recruitment, as the introduction of glutamic acid may disrupt the proper folding near the SIM and results in its inactivation, or that glutamic acid fails to fully mimic the steric and electrostatic properties of phosphorylation at these sites.

In sum, we have mapped a number of phosphorylation sites on PML. An examination of these, as well as a number of other reported sites in the literature, revealed that only those in the phospho-SIM motif appear to play a significant role in either the ability of PML to alter ND10 morphology or formation or in its response to viral infection (Table 2).

**Table 2:** Properties of PML-I and PML-I mutants.

	Sp100 Recruitment		Daxx Recruitment		SUMOylation	ICP0 Colocalization		Resistance to ICP0-Induced Degradation	Incoming Genome Recruitment
	A549	HepaRG	A549	HepaRG		A549	HepaRG		
**endogenous PML**	++	+++	ND	++	ND	+++	+++	ND	+++
**PML-I**	+	++	-	-	++	+++	+++	-	+++
**K65/160/490R**	++	-	+	-	-	+++	+++	-	+++
**S8;T28;S36/38/40A**	++	++	+	+	++	+++	+	-	+++
**S8;T28;S36/38/40E**	++	ND	+	ND	++	+++	ND	-	+++
**S117A**	++	++	+	-	++	+++	+++	-	+++
**S117E**	+++	ND	++	ND	++	+++	ND	-	+++
**S480;T482;S493A**	++	++	-	-	++	+++	+++	-	+++
**S480;T482;S493E**	++	ND	+	ND	++	+++	ND	+	+++
**S504/505A**	++	++	+	+	++	+++	+++	+	+++
**S504/505E**	++	ND	-	ND	++	+++	ND	+	+++
**S518A**	++	++	-	+	+++	+++	+++	-	+++
**S518E**	++	ND	+	ND	++	+++	ND	+	+++
**S527/530A**	+	++	-	++	++	+++	+++	-	+++
**S527/530E**	+	ND	-	ND	++	+++	ND	-	+++
**S560/561/562/565A**	++	+++	++	+++	+	+++	+++	-	-
**S560/561/562/565E**	++	ND	++	ND	++	+++	ND	-	-
**S565A**	ND	+++	ND	+++	+	+++	+++	-	+++
**S565E**	++	ND	++	ND	++	+++	ND	-	+++
**V556/557/558A;I559S**	+++	+++	+++	+++	+	+++	+++	ND	-
**ΔNLS**	-	ND	+	ND	-	+++	++	++	-

PML-I and the PML-I mutants were scored for their relative ability to recruit Sp100 or Daxx, their levels of SUMOylation, their ability to colocalize with ICP0, their resistance to ICP0-mediated degradation, their recruitment to incoming viral genomes, and ability to suppress viral plaque formation as detailed in Figure 1, Figure 2, Figure 3, Figure 4, Figure 5, Figure 6 and Figure 7, Figure S1-S5. ND, not determined; -, unable; +, weak; ++, moderate; +++, strong.

## Supplementary Materials

Supplementary materials can be accessed at: http://www.mdpi.com/2073-4409/3/4/1131/s1.
